# Impregnation of Activated Carbon with Organic Phase-Change Material

**DOI:** 10.3390/ma17010067

**Published:** 2023-12-22

**Authors:** Jiyeol Bae, Suho Kim, Kwangsoo Kim, Soyoung Baek

**Affiliations:** 1 Department of Environment Research, Korea Institute of Civil engineering and Building Technology, Goyang-si 10223, Republic of Korea; baejiyeol@kict.re.kr (J.B.); kskim@kict.re.kr (K.K.); 2 Department of Civil and Environmental Engineering, University of Science and Technology (UST), Daejeon 34113, Republic of Korea; 3 Construction Test & Assessment Center, Korea Institute of Civil engineering and Building Technology, Goyang-si 10223, Republic of Korea; suhokim@kict.re.kr

**Keywords:** phase-change material, latent heat, activated carbon, energy storage

## Abstract

In this study, we developed a thermal storage medium comprising porous activated carbon filled with organic phase-change materials (PCMs) that utilizes the latent heat of phase-change to absorb heat during heating and release heat during cooling. For the activated carbon, we used both charcoal-based powdered activated carbon (250–350 mesh) and granular activated carbon. The organic phase-change materials used in the experiments were dodecane, tridecane, tetradecane, and pentadecane. Material properties such as thermal conductivity, latent heat, and melting temperature range were evaluated experimentally and theoretically, with the results observed to be consistent. The cyclic thermal performance of the proposed medium was also evaluated. Notably, filling the activated carbon with a mixture of organic PCMs resulted in the highest temperature-moderating effect. The procedure and results presented in this study are expected to aid in further improvement in the performance of thermal storage media containing PCM where stable temperatures are required, including building heating and cooling.

## 1. Introduction

The depletion of fossil fuels has made the utilization of alternative energy sources a global imperative for survival [1,2]. Consequently, renewable energy sources such as solar heat, solar power, and wind energy are being researched as viable substitutes for fossil fuels [3,4]. However, the infrastructure required to convert these energy sources into electricity means that they have limitations as commercial power sources [5]. For example, it is costly to overcome challenges such as variable energy density and unpredictable generation frequency. Rather than building large-scale power generation facilities with high initial investment and low energy efficiency, thermal storage systems are attracting attention [5,6,7]. Among them, thermal storage systems utilizing phase-change materials (PCMs) are particularly promising, offering non-polluting, sustainable solutions with high thermal storage density [8,9].

PCMs possess high latent heat capacities and can accumulate or release heat during transitions from one state to another, such as from solid to liquid or liquid to gas [10,11]. Using latent heat associated with phase changes allows for smaller volumes of thermal storage media and spaces, while maintaining the capability to release or absorb heat at a constant temperature [12,13]. PCMs have various phase transition temperatures and heats of fusion, depending on the particular material, and thus they can be used efficiently for specific applications [14,15]. Their application scope ranges from solar thermal heating systems to textiles, insulating materials, and building materials [16]. Researchers have explored the integration of PCMs into solar thermal systems to store excess solar energy during the day for later use [17,18]. PCM-enhanced building materials have been investigated for stabilizing indoor temperatures by absorbing and releasing heat during the day–night cycle. PCMs store energy as latent heat when they transition between states, and in general the relatively small volume change that occurs during solid-to-liquid phase transitions is utilized [19]. However, there are several challenges to their application in various industries. One major challenge is the leakage of the PCM and a decrease in the effectiveness of the system. At this point, an “encapsulation” process is needed to stabilize the liquid state of the PCM [20]. The materials used for encapsulation are typically polymers, and various processing steps are required depending on the method used to contain the PCM [21]. The chemical contaminants and energy required for this process offset the various advantages of PCMs, hindering their commercialization [22]. Thus, encapsulation processes should not add significantly to the overall cost of the PCM system, especially for applications like building materials or consumer electronics. The environmental impact of both the encapsulation material and the encapsulation process itself is also a consideration. Therefore, if an environmentally friendly non-polluting encapsulation process were to be established, PCMs could become effective thermal storage materials that can compensate for the limitations of existing alternative energy sources [23,24].

The aim of this study was to develop a simpler and more effective thermal storage medium using activated carbon as a container for PCMs. The objective was to facilitate encapsulation by devising a method to fill the pores of activated carbon with organic PCMs, leveraging the principle of capillary adsorption. We also aimed to provide a more accurate and quantitative analysis of PCM-filled activated carbon based on thermodynamic modeling.

## 2. Materials and Methods

### 2.1. Materials

The activated carbon used in this study was supplied by YAKURI Pure Chemical (Kyoto, Japan) and was prepared using cypress wood as the starting material. We used powdered activated carbon (250–350 mesh), which we refer to hereinafter as PW-AC (Powdered Activated carbon), and granular activated carbon manufactured from the same material, hereinafter referred to as GW-AC (Granular Activated carbon). The properties of the activated carbon used to encapsulate the PCM are listed in Table 1. A total of four types of PCMs were used in the experiment, all of which were alkanes: dodecane, tridecane, tetradecane, and pentadecane.

### 2.2. Method of Filling Activated Carbon with Organic PCM

**Figure 1:**
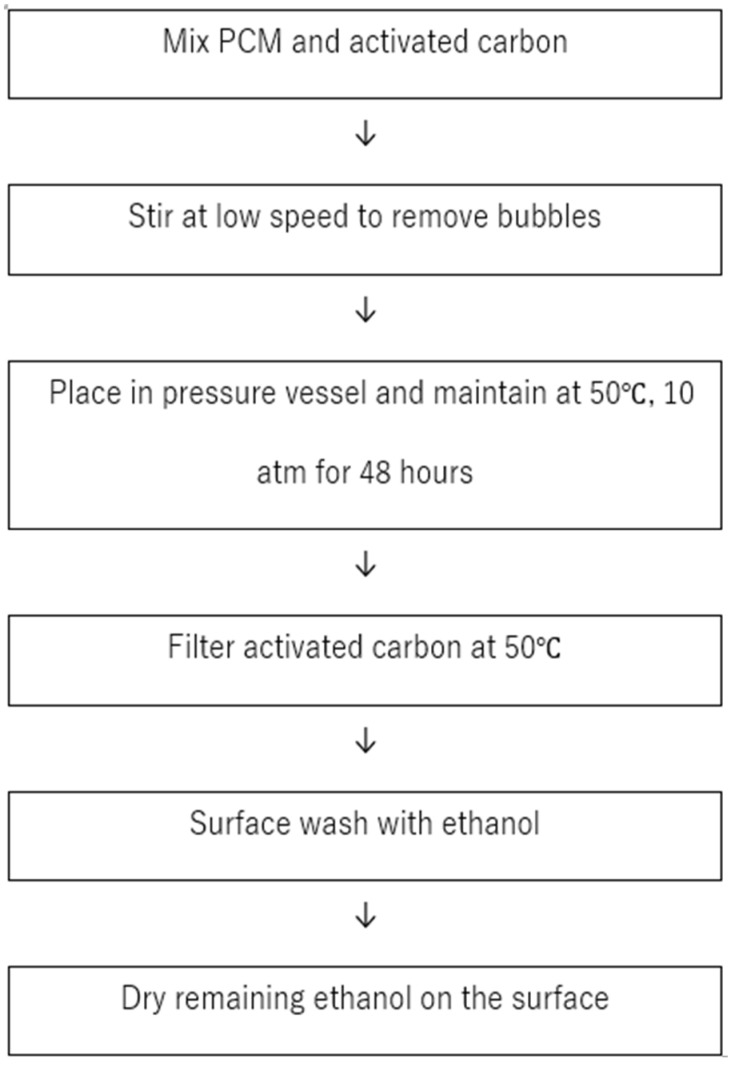
Procedure for filling activated carbon with organic PCM.

Figure 1 illustrates the method used to fill the pores of activated carbon with organic PCMs. A liquid form of the organic PCM is placed in a beaker and activated carbon is added while stirring. The stirring is performed at a low speed until no bubbles remain in the mixture in the beaker. The beaker is then placed in a pressure vessel, and using a temperature-controlled heating element and nitrogen gas, the temperature and pressure inside the vessel are maintained at 50 °C and 10 atm, respectively, for 48 h. Maintaining the temperature at 50 °C during the filling process ensures that the PCM is not too viscous, which eases the filling process. Moreover, this promotes consistent adsorption under isothermal conditions, ensuring that the filling process is reproducible. At 50 °C, the PCM fills the pores of the activated carbon through adsorption and capillary action. It is believed that the PCM is also adsorbed onto the surface of the activated carbon. Unlike the PCM in the pores, the material on the surface tends to leak during the solid-to-liquid transition; therefore, it needs to be washed off during the manufacturing process. Ethanol was chosen to wash the surface because it evaporates easily, and because the solubility of the organic PCMs in ethanol is low, this solvent is unlikely to disturb the material within the pores. A minimal amount of ethanol was applied to rinse the surface of the filtered activated carbon, and any remaining ethanol was allowed to evaporate at room temperature.

### 2.3. Temperature Measurement Devices

The temperature measurement setup consists of a glass container to hold the PCM-filled activated carbon, an insulating material to induce one-dimensional heat flow through the bottom of the glass container, a temperature gauge for measuring heat absorption and release, and a temperature-controlled chamber.

**Figure 2:**
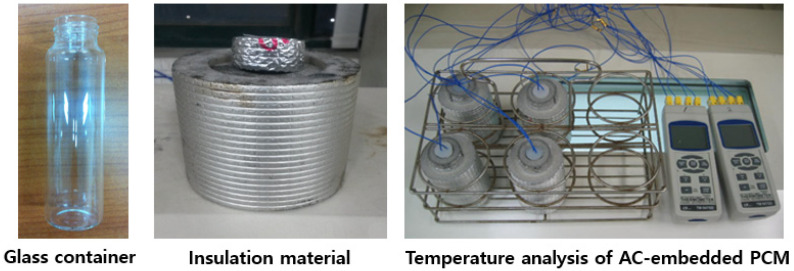
Experimental setup for measurement of the temperature of AC-embedded PCM.

The activated carbon filled with both organic and inorganic PCMs was packed into the glass container shown in Figure 2 by tapping it 30 times from a height of approximately 3 cm to ensure uniform compaction. Since the material is a particulate substance with pores, its thermal conductivity varies significantly with the degree of compaction and porosity. Therefore, all the activated carbon samples were compacted in the glass container using the same method. The dimensions of the glass container are as follows: outer diameter, 30.0 mm; height, 108 mm; side thickness, 1.2 mm; and bottom thickness, 1.0 mm. The glass container holding the PCM-filled activated carbon was placed in the insulating material, as shown in Figure 2, to guide the thermal energy to through the bottom of the glass container. The insulating layer is made from polyurethane foam and has an outer diameter of approximately 10 cm. The bottom is open, allowing heat conduction due to the temperature difference between the ambient temperature and the temperature inside the activated carbon. The position of the measurement sensor greatly influences the results; therefore, all the sensors were positioned 1 cm above the bottom of the glass container. The temperature sensor was programmed to measure and store the temperature every 10 s. The temperature-controlled chamber used in this experiment (TC-ME-065, JEIO TECH, Daejeon, Republic of Korea) has an internal volume of 40 L and its internal temperature is maintained at the set value through forced convection. For thermodynamic analysis, a commercial laser flash apparatus instrument (LFA467, NETZSCH, Bayern, Germany) was used as the effective thermal conductivity measurement system. Given the nature of powdered samples, the density can easily change depending on the degree of compaction, so measurements were acquired with great care. The latent heat of fusion of the PCM-filled activated carbon was analyzed using differential scanning calorimetry (DSC) techniques (Q1000, TA Instruments, New Castle, DE, USA).

### 2.4. Analytical Instrumentation

The latent heat of fusion of the PCM-filled activated carbon was analyzed using differential scanning calorimetry (DSC) techniques (Q1000, TA Instruments). N2 adsorption/desorption experiments for pore properties of sample were carried out using a Micromeritics ASAP 2010 volumetric adsorption analyzer (Norcross, GA, USA).

## 3. Results and Discussion

### 3.1. Measurement and Prediction of Latent Heat for Activated Carbon/PCM Mixtures

**Figure 3:**
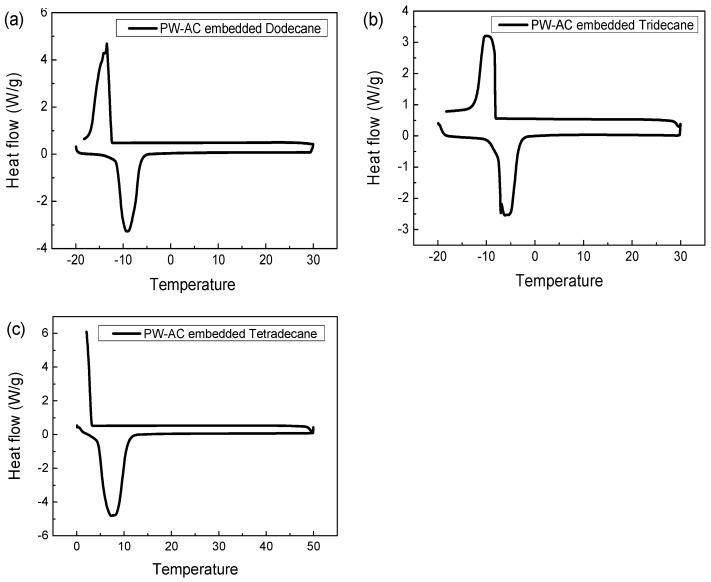
DSC measurement results for powdered activated carbon filled with three different organic PCMs: (**a**) activated carbon/dodecane, (**b**) activated carbon/tridecane, (**c**) activated carbon/tetradecane.

While the latent heats of pure PCMs have been widely reported, there is very limited information on mixtures of PCM and activated carbon for thermal storage [25]. Accordingly, we analyzed the latent heat of fusion for activated carbon/PCM mixtures using differential scanning calorimetry (DSC) techniques. DSC can be used to analyze the physical and chemical properties of a sample by interpreting temperature and heat flow changes from the energy applied to the sample. Quantitative information such as melting point and melting temperature range can be obtained from the position, shape, and number of peaks in the resulting differential thermogram, and the latent heat of fusion can be quantitatively determined by integrating the area under the peaks [26]. Figure 3 presents the differential thermograms of powdered activated carbon filled with three different types of organic PCMs, dodecane, tridecane, and tetradecane.

In Figure 3, it was confirmed that AC embedded with dodecane, tridecane, and tetradecane began crystallization and melting at −10, −5, and 5 degrees, respectively. These temperatures are almost the same as the generally known phase transition temperatures of dodecane, tridecane, and tetradecane. This means that there is almost no change in the thermal properties of the phase-change material impregnated with activated carbon, and it was confirmed that the phase-change material maintains its inherent thermal properties even when the phase-change material is adsorbed within the pores.

The thermal conductivity of the three types of activated carbon/organic PCM mixtures was calculated using effective medium theory. Calculations were made using both the Maxwell Garnett (MG) model and the Bruggemann model, considering the characteristics of the activated carbon/PCM materials, while effects due to nanopore layer formation in the PCM and convection were not considered separately [27].

To apply effective medium theory, it is also important to measure the thermal conductivity of activated carbon itself [28,29]. Despite several existing reports, thermal property measurements remain limited for porous activated carbon, even compared to various pure organic/inorganic phase-change materials. The challenge in measuring the thermal properties of activated carbon arises because the size of the activated carbon particles varies, and the packing density also affects the measured thermal properties [30,31].

Conventional measurements of the thermal conductivity of activated carbon can be broadly categorized into methods that generate a one-dimensional (1-D) heat flow to measure temperature distribution and those that use the transient hot wire measurement technique [31,32]. Menard et al. measured the thermal conductivity of granular packed coconut-based activated carbon (PICA, Paris, France) to be approximately 0.15 W/mK using a 1-D cylindrical test bed filled with the material and thermocouples for temperature distribution measurements. However, no information was provided in the report of this study regarding the actual particle size distribution or packing density of the activated carbon [33]. Chen et al. used the transient hot wire measurement technique to obtain the thermal conductivity of activated carbon with a particle size range of 20–50 mesh (200–840 micron) [28]. Commercially available hot wire equipment (TC3020, XIATECH, London, UK) was used, and thermal conductivities ranging between 0.143 and 0.165 W/mK were measured. Harder et al. measured the thermal conductivity of activated carbon sifted through an approx. 40 µm mesh using the hot disk technique and obtained a value of about 0.43 W/mK [34].

These existing studies suggest that the thermal conductivity of activated carbon can vary significantly depending on particle size or packing density. In this study, the thermal conductivity of the activated carbon was estimated to be 0.43 W/mK. Based on this, the properties of the activated carbon/PCM mixture was predicted using both the MG and Bruggeman models, and the results are summarized in Table 2. Both models predicted similar effective thermal conductivity values, and the thermal conductivities of the three types of organic PCM encapsulated in activated carbon in the experiment were predicted to be very similar.

### 3.2. Dynamic Model of Activated Carbon/PCM Mixture

**Figure 4:**
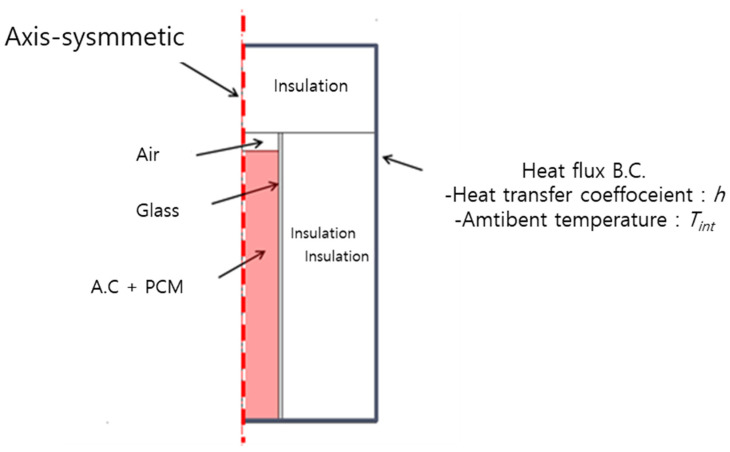
Computational domain and boundary conditions used in the modeling.

To analyze the thermal characteristics of the activated carbon/PCM mixture, we developed a dynamic thermal model. As shown in Figure 4, a two-dimensional (2-D) axisymmetric model, allowing for the analysis of cylindrical samples similar to the experimental setup, was adopted. It includes a glass container, holding the PCM-filled activated carbon, and surrounding insulation. The filling height and other geometric parameters related to the PCM-filled activated carbon were identical to those in the experiment.

Using this defined computational domain, the thermal characteristics of the system were analyzed based on the unsteady energy equation:

(1) $$\rho C_{p}\frac{\partial T}{\partial t}+\nabla \cdot (-k\nabla T)=Q,$$

where *Q* represents the heat exchanged between the system and the external environment at the boundary, *ρ* is the sample density, *C_p_* is the specific heat capacity of the sample, *T* is temperature, and *k* is the Boltzmann constant. This energy equation was numerically solved by subdividing the computational domain into multiple small grids, and a numerical analysis technique using the finite element method was applied. The material properties of the PCM-filled activated carbon, as defined using Equation (1), were determined using the previously mentioned effective thermal conductivity and effective specific heat models (see Section 3.1), utilizing the mass and volume ratios of the PCM-filled activated carbon. For the boundary conditions, the heat flow boundary condition outside the insulation was defined as a function of the convective heat transfer coefficient and ambient temperature. For the ambient temperature, time-dependent temperature changes measured in the experimental chamber were used as inputs for the model. To estimate the convective heat transfer coefficient within the chamber, we compared the analysis results with the temperature increases as measured during an experiment using water and applied a coefficient of 100 W/(m^2^K).

### 3.3. Characteristics of Activated Carbon before and after Organic PCM Filling

To confirm the weight ratio of activated carbon filled with the PCMs, the weight change for AC before and after PCM loading was measured. The experiment was conducted using about 50 g of activated carbon, and the results are shown in Table 3. In the case of powdered activated carbon, the weight increased by about 2.19 g after tridecane filling, and granular activated carbon increased by about 1.54 g after tridecane filling. Based on this, the PCM content in activated carbon was confirmed to be 4.18% and 2.99% for powdered activated carbon and granular activated carbon, respectively.

The specific surface areas of the activated carbon samples filled with the organic PCMs are listed in Table 3. As shown in the table, the specific surface areas of the powdered activated carbon (PW-AC) and granulated activated carbon (GW-AC) were found to be 20.4 and 2.8 m^2^/g, respectively. A significant reduction in pore volume was also observed after the activated carbon samples had been filled with PCM; because this decrease was most probably owing to the fact that the PCM filling clogged the pores of the activated carbon, we can conclude that the micro-pores of the activated carbon had been successfully filled with the PCM [35,36]. However, the average pore size for both the powdered and granulated activated carbon increased from 23.6013 to 155.5545 Å and from 42.4180 Å to 57.0679 Å, respectively. This suggests that the filling primarily occurred in smaller pores, which we attribute to the micro-pores [37].

### 3.4. Thermal Absorption and Release of Alkane PCM Embedded in Activated Carbon

**Figure 5:**
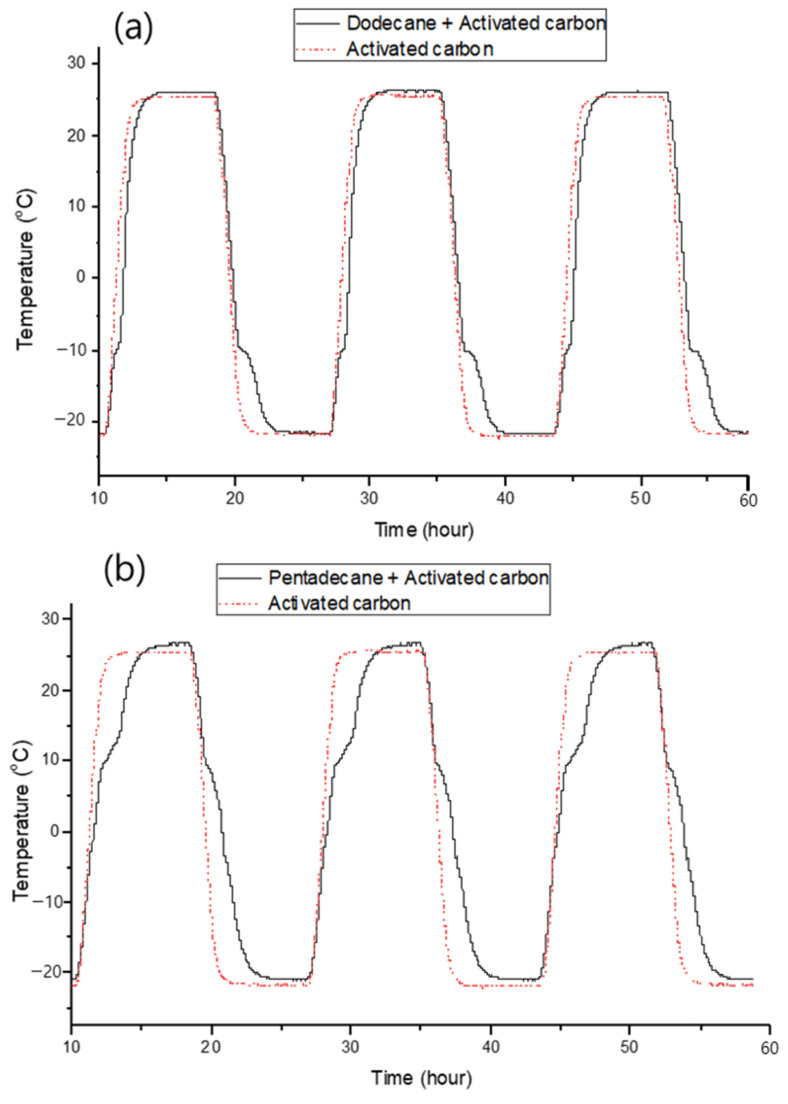

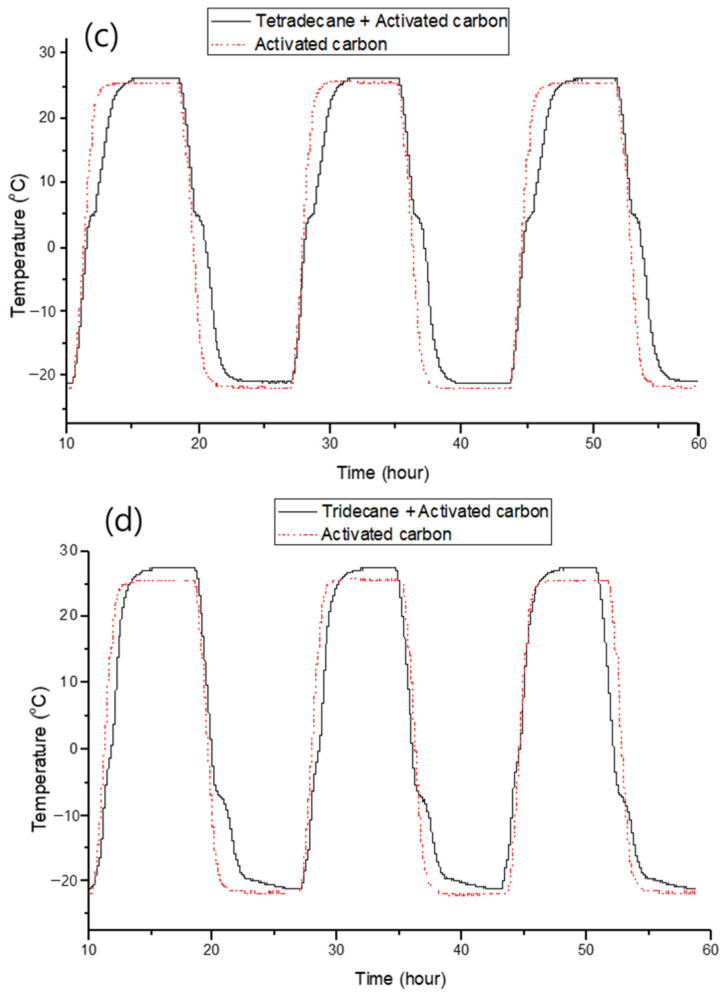
Thermal absorption and release of activated carbon filled with alkane PCMs: (**a**) dodecane, (**b**) tridecane, (**c**) tetradecane, and (**d**) pentadecane.

Figure 5 shows the thermal absorption and release curves of the activated carbon filled with the alkane PCMs. We observed that each PCM exhibited delays in thermal rise and fall at their respective phase-change temperatures (dodecane: −10 °C, tridecane: −5 °C, tetradecane: 5 °C, pentadecane: 10 °C). This indicates that the high-pressure filling method does not affect the thermal characteristics of the PCM and is an efficient approach. After three repeated experiments, we demonstrated that the activated carbon consistently maintained its thermal absorption and release capabilities, further demonstrating that it is an effective support material for PCMs.

### 3.5. Thermal Stability of Activated Carbon Filled with PCM

**Figure 6:**
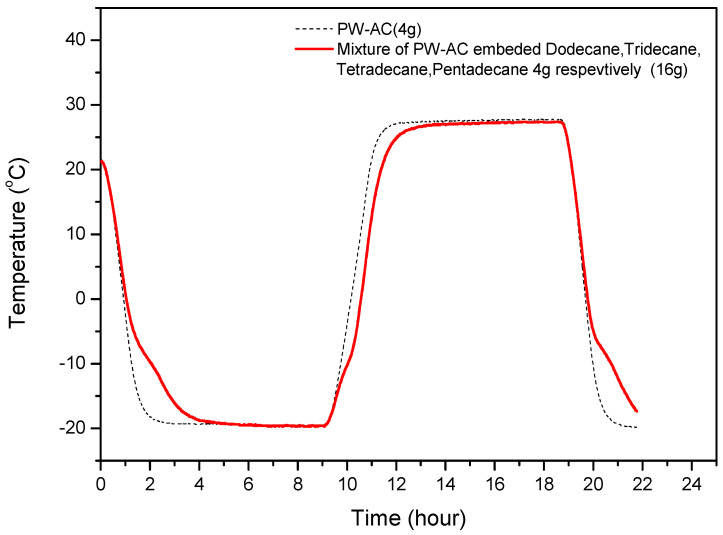
Temperature changes in samples of activated carbon filled with a low-temperature PCM mixture (4 g each of dodecane, tridecane, tetradecane, and pentadecane).

Figure 6 presents the results of the evaluation of the thermal absorption and release characteristics of powdered activated carbon filled with a mixture of 4 g of each of four alkane organic PCMs (dodecane, tridecane, tetradecane, and pentadecane). The amount of activated carbon used in this study was approximately 50 g. The amount of phase-change material that can be filled in the pores of 50 g of activated carbon was determined to be approximately 3.8 to 4.5. In order to distinguish the characteristics of phase-change materials, the amount was unified to 4 g. The measurement results showed that although the low-temperature PCMs have different melting points, they do not exhibit independent melting points when mixed together. Notably, high latent heat characteristics were observed at −10 °C. Among the four types of PCMs, the filling capacity per unit weight of activated carbon was highest for dodecane, which has a phase-change temperature of approximately −10 °C. This is because dodecane also has the highest latent heat per unit mass. However, minor inflection points in other temperature ranges suggest that latent heat from tetradecane and pentadecane is also being generated simultaneously. This is attributed to the low thermal conductivity of activated carbon, which delays the transmission of the thermal absorption and release to the temperature measurement point. The results in Figure 4 suggest that temperature changes from the various PCMs occur sequentially, resulting in observed delays in the temperature rise and fall across a broad temperature range, rather than a specific one.

**Figure 7:**
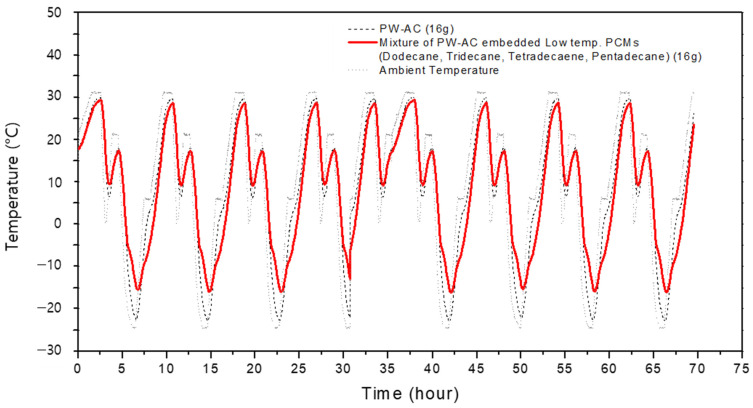
Long-term temperature changes, recorded over 70 h, in samples of activated carbon filled with a low-temperature PCM mixture (4 g each of dodecane, tridecane, tetradecane, pentadecane).

Figure 7 shows temperature measurement results for activated carbon filled with a mixture of alkane organic PCMs at various ambient temperatures. The gray dotted line shows the ambient temperature set using a temperature control chamber, while the black dotted line indicates the temperature of powdered activated carbon that had not been filled with PCM. The bold solid line indicates the temperature of the powdered activated carbon filled with the mixture of four types of alkane PCMs. The experiment was conducted continuously over a period of 70 h, with external temperatures varying widely from −20 to 30 °C. No degradation in terms of temperature or time is detectable on the graph. The phase-change temperatures for the four types of alkane PCMs are within the range of −15 °C to 15 °C, so their buffering effect on the ambient temperature was more pronounced when the ambient temperature was below 15 °C rather than above it. Comparing the temperature change range, it was confirmed that the activated carbon filled with PCM had a temperature-moderating effect as its temperature change range was up to 10 °C lower than that of the un-filled activated carbon.

### 3.6. Reproducibility of Low-Temperature PCM Characteristics after Long-Term Exposure to High Temperatures

**Figure 8:**
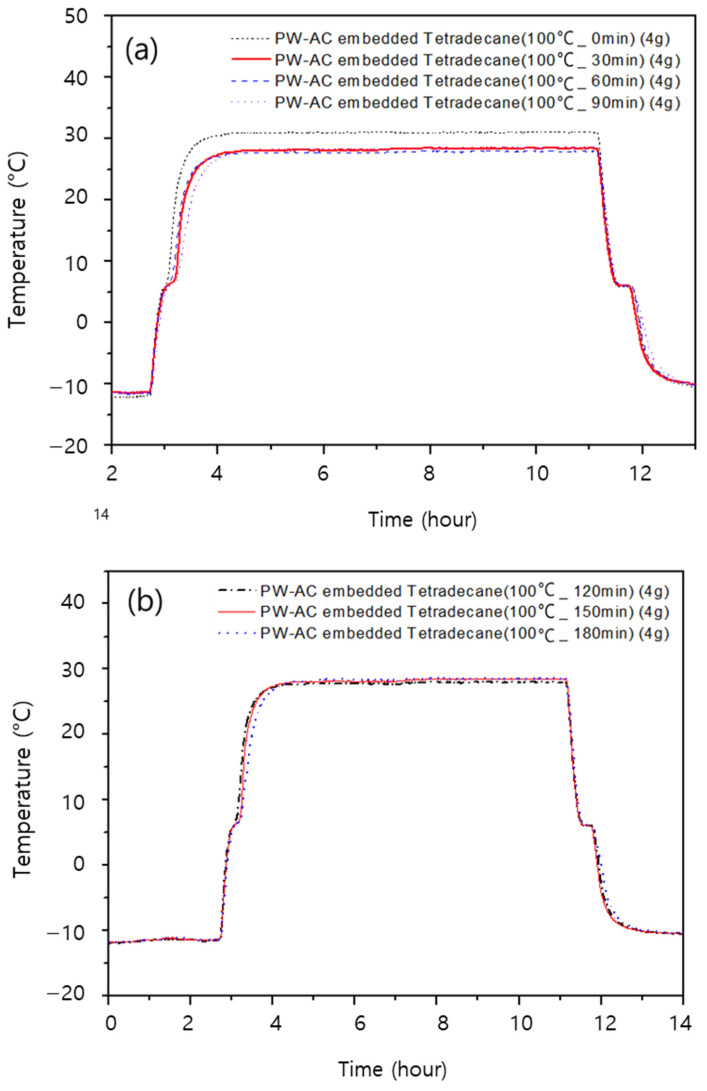
Temperature changes in samples of activated carbon filled with tetradecane extracted at 30 min intervals from storage at 100 °C: (**a**) samples stored for 30, 60, 90 min and (**b**) samples stored for 120, 150, and 180 min.

To evaluate the thermal stability of the PCM-filled activated carbon, we used samples of powdered activated carbon filled with tetradecane and stored them in a 100 °C oven for 30, 60, 90, 120, 150, and 180 min. We then observed their thermal absorption and release behavior in response to the changing ambient temperature. The results are presented in Figure 8. We confirmed that the phase change for tetradecane occurred stably at its phase-change temperature of 5 °C, even when the ambient temperature varied between −10 °C and 30 °C. This confirms that the PCM remained stably contained within the activated carbon, even at a high temperature of 100 °C, without any leakage.

### 3.7. Thermodynamic Analysis

**Figure 9:**
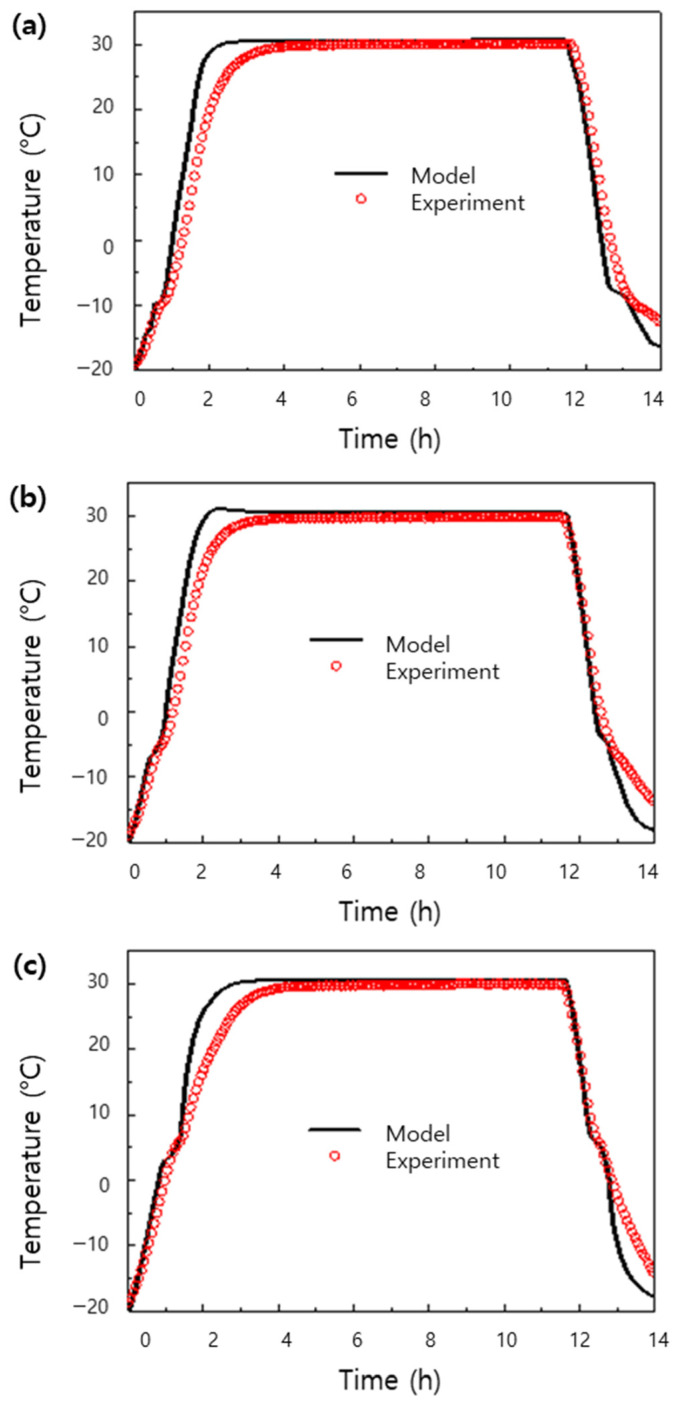
Comparison of results from experiments and models for thermal absorption and release of PCMs embedded in the pores of activated carbon: (**a**) activated carbon filled with dodecane, (**b**) activated carbon filled with tridecane, and (**c**) activated carbon filled with tetradecane.

Figure 9 presents the results of experiments on the transient thermal storage characteristics of mixtures of activated carbon and dodecane, tridecane, and tetradecane, along with the results of calculations based on a model constructed using the same conditions. The calculated and experimental results were found to be in quite good agreement, and the proposed model accurately replicated and predicted phase-change characteristics within the phase-change temperature range. However, there was some discrepancy in the rate of temperature rise between the experimental and calculated results, and the model can be improved by using more accurate thermal property measurements considering factors such as the packing density [38]. Using such a dynamic model, it is possible to predict not only the thermal storage capacity but also various parameters such as the thermal storage and release rates for thermal storage systems operating across different temperature ranges, and hence this model should be helpful for system design and optimization [39].

Compared to the unfilled activated carbon, the thermal storage capacity was significantly enhanced for the PCM-filled activated carbon, owing to the latent heat of fusion of the PCMs. The ratio between the thermal storage for the filled and unfilled activated carbon, *R*, was calculated using Equation (2), and this ratio varies with the operating temperature range of the system:

(2) $$R=\frac{\int_{T_{1}}^{T_{2}}C_{p,eff,AC+PCM}(T)dT}{\int_{T_{1}}^{T_{2}}C_{p,AC}(T)dT}.$$

Table 4 lists these ratios for various activated carbon/PCM samples and activated carbon samples of the same mass, for the external temperature rise from −20 °C to 30 °C. The ratio of thermal storage capacity increase was highest for dodecane at 3.67, followed by tetradecane (3.65) and tridecane (2.92). This can be explained in the sense that the larger the material’s thermal storage capacity, the less the temperature changes and the better it is at accumulating heat energy [40].

## 4. Conclusions

In conclusion, this research investigated the use of activated carbon as a container for phase-change materials (PCMs) to enhance thermal storage efficiency. The study addressed the lack of information on specific activated carbon/PCM mixtures, explored their thermodynamic properties using differential scanning calorimetry (DSC), and predicted thermal behavior with effective medium theory. A dynamic thermal model was developed to analyze heat transfer characteristics, demonstrating the system’s efficient heat absorption and release capabilities. Physical changes in activated carbon after PCM filling were observed, with a reduction in specific surface area indicating successful PCM filling in micro-pores. The study confirmed the thermal stability of PCM-filled activated carbon, even after exposure to high temperatures, highlighting its suitability for long-term use. The research also quantified the enhanced thermal storage capacity of PCM-filled activated carbon compared to unfilled carbon. This study lays the foundation for efficient thermal storage systems using activated carbon and PCMs. This novel thermal storage medium could be used for industrial applications, including the fabrication of building materials that involve cyclical heating and cooling, helping to optimize energy consumption and reduce operating costs. Thus, using activated carbon as an encapsulation medium for PCMs presents sustainability with a higher ecological footprint and a lower environmental impact during the manufacturing phase. Future research should focus on refining the dynamic model, considering factors like packing density, and exploring real-world applications. Thus, future study could investigate the scalability for widespread adoption and economic viability of this technology for use in renewable energy solutions.

## Figures and Tables

**Table 1:** Properties of activated carbon used for encapsulating PCMs.

Properties	Activated Carbon	
	PW-AC (Powdered)	GW-AC (Granular)
Mesh	250–350	8–23
Particle size	Approximately 40 μm	Approximately 1570 μm
BET surface area	1039.1472 m^2^/g	1060.1281 m^2^/g
Pore volume	0.6131 cm^3^/g	1.1242 cm^3^/g
Pore size	23.6013 Å	42.4180 Å

**Table 2:** Properties of PCMs and activated carbon used in the experiment.

		Phase Transient Temperature (°C)	Latent Heat (kJ/kg)	Specific Gravity	Classification for Usage
Organic Phase-Change Material	Dodecane	−10	349.3	0.78	Low temp
	Tridecane	−5	377.7	0.75	Low temp
	Tetradecane	5	124.9	0.76	Low temp
	Pentadecane	10	163	0.77	Low temp

**Table 3:** BET analysis results for activated carbon before and after PCM filling.

		PW-AC		GW-AC	
		PW-AC	PW-AC-Embedded Tridecane	GW-AC	GW-AC-Embedded Tridecane
Weight change (g)		50.23	52.42	50.10	51.64
BET analysis results	Surface area [m^2^/g]	1039	20.4	1060	2.8
	Single point adsorption total pore volume of pores [cm^3^/g]	0.61	0.07	1.12	0.004
	Adsorption average pore width [Å]	23.6013	155.5545	42.4180	57.0679

**Table 4:** Ratios between thermal storage capacities of activated carbon/PCM and pure activated carbon (temperature increase range: −20 to 30 °C).

Sample	Thermal Storage Capacity Increase Ratio *R* QAC+PCM/QAC
AC + Dodecane	3.67
AC + Tridecane	2.92
AC + Tetradecane	3.65

## Data Availability

The data presented in this study are available on request from the corresponding author.
